# Influence of different types of sterile cytoplasms (A3, A4, 9E) on the combining ability of CMS lines of sorghum

**DOI:** 10.18699/VJ20.648

**Published:** 2020-10

**Authors:** O.P. Kibalnik, L.A. Elkonin

**Affiliations:** Russian Research and Project-technological Institute of Sorghum and Maize, Saratov, Russia; Agricultural Research Institute of the South-East Region of Russia, Saratov, Russia

**Keywords:** Sorghum bicolor (L.) Moench, cytoplasmic male sterility, heterosis, combining ability, cytoplasmic effects, drought, Sorghum bicolor (L.) Moench, цитоплазматическая мужская стерильность, гетерозис, комбинационная способность, цитоплазматические эффекты, засуха

## Abstract

Investigation of the effect of the cytoplasm on the combining ability (CA) of lines with cytoplasmic male sterility (CMS) is of considerable interest in terms of understanding the genetic functions of the cytoplasm and for practical purposes to create hybrids with improved economically valuable traits. In order to investigate the effect of different types of sterile cytoplasm (A3, A4, 9E) on CA in sorghum, we studied the manifestation of a number of biological and agronomic traits in 54 F1 hybrid combinations obtained using iso-nuclear CMS lines with the nuclear genome of the line Zheltozernoye 10, differing only in the types of sterile cytoplasm (A3, A4 and 9E). Eighteen varieties and lines of grain sorghum developed at the Russian Research and Project-technological Institute of Sorghum and Maize were used as paternal parents. The CA was determined by the topcross method. F1 hybrids and their parents were grown in 2015–2017 in conditions of insufficient (2015–2016: HTC (hydro-thermal coefficient) = 0.32–0.66), or good water availability conditions (2017: HTC = 1.00). On average, for three years of testing, a positive effect of the 9E cytoplasm on the general combining ability (GCA) (0.63) and negative effects of the A3 and A4 cytoplasms (–0.32 and –0.31) for the inflorescence length were noted. In dry seasons, significant positive effects of the 9E cytoplasm on GCA for the length of the largest leaf, and positive effects of the A3 cytoplasm on GCA for the plant height, and negative effects of the A4 cytoplasm on GCA for these traits were observed. No differences were observed during the wet season. The type of CMS did not affect the GCA for the width of the largest leaf and grain yield. The dispersion of specific combining ability (SCA) in the dry seasons was significant for the following traits: leaf length, plant height, panicle length and width, and grain yield, the 9E cytoplasm had the highest SCA dispersion, whereas the A4 cytoplasm had the smallest one. The data obtained indicate that different types of sterile cytoplasm of sorghum make a different contribution to CA under conditions of drought stress.

## Abstract (ru)

Изучение влияния цитоплазмы на комбинационную способность (КС) линий с цитоплазмати- ческой мужской стерильностью (ЦМС) представляет значительный интерес в плане понимания генетиче- ских функций цитоплазмы у растений и в практических целях для создания гибридов с улучшенными хозяй- ственно ценными признаками. С целью выяснения характера влияния разных типов стерильных цитоплазм (А3, А4, 9Е) на КС у сорго исследовали проявление ряда агрономически ценных признаков у 54 гибридных комбинаций F1, полученных с использованием в качестве материнских родителей изоядерных ЦМС-линий, созданных на основе линии Желтозерное 10 и различающихся только типами стерильных цитоплазм (А3, А4 и 9Е). В качестве отцовских родителей были 18 сортов и линий зернового сорго селекции Российского НИИ сорго и кукурузы. Комбинационную способность определяли методом топкросса. Родительские компонен- ты и гибриды F1 выращивали в 2015–2017 гг. в условиях недостаточной (2015–2016 гг.: гидротермический коэффициент (ГТК) = 0.32–0.66) либо хорошей (2017 г.: ГТК = 1.00) влагообеспеченности. В среднем за три года испытаний выявлены положительное влияние цитоплазмы 9Е на общую комбинационную способность (ОКС) по длине соцветия (0.63) и отрицательные эффекты цитоплазм А3 и А4 (–0.32 и –0.31) на ОКС по этому признаку. В засушливые сезоны отмечены значимые положительные эффекты цитоплазмы 9Е на ОКС по дли- не наибольшего листа, цитоплазмы А3 – на ОКС по высоте растений и отрицательное влияние цитоплазмы А4 на эти признаки. Во влажный сезон различия отсутствовали. Тип ЦМС не оказывал влияния на ОКС по ширине наибольшего листа и урожайности зерна. Дисперсия специфической комбинационной способности (СКС) в засушливые сезоны оказалась значимой для следующих признаков: длина листа, высота растений, длина и ширина метелки, урожайность. При этом линия с цитоплазмой 9Е отличалась наиболее высокими показателями дисперсии СКС, тогда как линия с цитоплазмой А4 – наименьшими. Полученные данные сви- детельствуют, что разные типы стерильных цитоплазм сорго вносят различный вклад в КС в условиях засухи.

## Introduction

The cytoplasm as the environment for the functioning of the nuclear genes plays an important role in the genetic control of many plant traits. Along with the well-known, and in some cases well-studied mutations of variegation and cytoplasmic male sterility (CMS) that arise as a result of rearrangements in the chloroplast and mitochondrial genomes, there are many examples of the influence of the cytoplasmic environment on the manifestation of many plant traits, including those with important biological and economic value. This effect of the cytoplasm may be caused by retrograde regulation of nuclear gene expression by signals produced by cytoplasmic organelles under the influence of environmental factors (Fujii, Toriyama, 2008). The genetically different plastomes and mitochondrioms can respond differently to environmental signals and affect the expression of nuclear genes. In addition, the cytoplasm is capable of causing inherited changes in the nuclear genome by paramutations (Zavalishina, Tyrnov, 2003, 2010), and changing the methylation of nuclear gene sequences (Xu et al., 2013; Ba et al., 2014) including nucleotide sequences of mobile genetic elements (Elkonin et al., 2018), that can alter the expression level of nuclear genes and have significant genetic effects, since alteration of transposon methylation is one of the key factors of their mobility and, as a consequence, the occurrence of mutations (Yaakov, Kashkush, 2011).

Majority of agronomically valuable plant traits are polygenic and are formed as a result of the interaction of many nuclear genes among themselves and with environmental factors. In this regard, the cytoplasm can have a significant impact on the manifestation of these traits. There is a lot of data in the literature confirming the effect of the cytoplasm on agronomically valuable traits in wheat (Atienza et al., 2007), rice (Tao et al., 2011), cotton (Tuteja, Banga, 2011), pearl millet (Amiribehzadi et al., 2012), winter rye (Urban, Gordey, 2013), sorghum (Aruna et al., 2013), sunflower (Jan et al., 2014), maize (Kabanova et al., 2015), and mustard (Chakrabarty et al., 2015). Assuming that the manifestation of heterosis in F_1_ hybrids is determined, in considerable extent, by the combining ability (CA) of maternal lines, investigation of the effect of cytoplasm on CA is of significant interest. However, there are few studies on the effect of cytoplasm on CA. In pearl millet, the A4 and A5 cytoplasms caused positive effect on grain yield in comparison with A1 cytoplasm (Chandra-Shekara et al., 2007; Pujiar et al., 2019). Tests of new CMS sources of sunflower (XA, E002-91A, PKU-2A, ARG-2A, ARG-3A, ARG-6A, DV-10A, PHIR-27A, PRUN- 29A) showed a positive effect of sterile cytoplasms E002-91A (Helianthus annuus), ARG-3A (H. argophyllus) and ARG-6A (H. argophyllus) on the combining ability of maternal lines in seed productivity compared to normal cytoplasm NC-41B (Tyagi, Dhillon, 2016). A similar effect of A4 and A8 cytoplasms on the overall combining ability of lines has been described in rice (Young, Virmani, 1990).

In sorghum, there are contradictory data in the literature. The positive effect of A2 cytoplasm on the general combining ability (GCA) of CMS lines for the duration of the seedlingflowering interphase period, grain yield, grain weight per panicle and 100 grains, in comparison with A1 cytoplasm, has been described (Kishan, Borikar, 1989; Ramesh et al., 2006; Reddy et al., 2007, 2009). On the contrary, the lack of effects of A1 and A2 cytoplasms on heterosis was reported (Williams-Alanís, Rodríguez-Herrera, 1994).

The aim of this work was to study the effect of different sterile cytoplasms (A3, A4, 9E) on CA in sorghum using isonuclear CMS lines that differ only in types of sterile cytoplasm.

## Materials and methods

To identify cytoplasmic effects on combining ability, we used the early maturing alloplasmic iso-nuclear CMS lines of grain sorghum (Sorghum bicolor (L.) Moench) (Elkonin et al., 1997). These lines were obtained by consecutive backcrosses of fertile line Zheltozernoye 10 (Z10) to CMS lines А3 Тх398, А4 Тх398, 9Е Тх398 (provided by Dr. K.F. Schertz, Texas Agricultural Experimental Station, USA), carrying cytoplasms of the following accessions: IS1112C (A3), IS7920C (A4), and IS17218 (9E). In this study, maternal plants from the BC18 were used. As a pollen parents, early maturing varieties – Perspectivnoye 1, Mercury, Ogonek, Avans, Fakel, Azart, Garant, Topaz, Volzhskoye 615, and mid-early maturing varieties and lines – Start, L-KSI 28/13, Kamelik, Geleofor, Kremovoye, Pishchevoye 614, Sarmat, Vostorg, Pishchevoye 35 were used (18 in total). These pollen parents differed in manifestation of agronomically valuable traits and characterized by high adaptive ability to agro-climatic conditions of the region (Kibalnik et al., 2010, 2017). F_1_ hybrids obtained using these pollinators were characterized by mid-early maturity (110–117 days to full maturity).

Pollen parents were grown under strict isolation (the panicles were isolated with parchment bags before flowering) for 8–25 generations. All pollinators were sterility maintainers for the studied types of CMS, with the exception of Perspectivnoye 1 and L-KSI 28/13, which are the restorers of fertility for A4 and 9E CMS and provided 80–100 % seed set in conditions of strict isolation with parchment bags (Kibalnik, Semin, 2018).

The following traits were analyzed: plant height; the length and width of the largest leaf, the length and width of the inflorescence, mass and number of grains per panicle, and grain yield. Since paternal parents were not universal fertility restorers, and the majority of the studied hybrids were male sterile, in order to register the traits associated with grain productivity, the open-pollinated panicles were used. As far as F_1_ hybrids were grown in experimental field among hundreds of thousands of fertile plants, free pollination ensured 100 % seed setting all panicles of the studied hybrids. This approach has already been used to study the grain yield of hybrids in A3 cytoplasm (Moran, Rooney, 2003).

F_1_ hybrids (54 in total) were sown in the experimental field of the Russian Research and Project-technological Institute of Sorghum and Maize; in 2015–2017 in the third decade of May. The soil of the experimental plot was represented by medium loamy southern chernozem. The humus content in the arable layer was 3.5 %, nitrification ability – 7.7 mg/ kg; phosphorus – 34.2–35.7 mg/kg, potassium (in a carbon ammonium extract) – 349–378 mg/kg. In each season, zonal sorghum cultivation technology was used that did not include artificial irrigation (Gorbunov et al., 2012). The predecessor is steam field. The plots (7.7 m^2^) were allocated randomly in three replications. The plant standing density was set manually (100 thousand plants per ha). Evaluation of traits and yield was carried out according to methodology of state testing of crops (Metods of State Variety…, 1989). The combining ability of lines was determined by the topcross method (Savchenko, 1973). For statistical analysis of the experimental data Agros 2.09 software was used (Martynov, 1999).

Weather conditions varied over the seasons of the study. The 2017 season was characterized by high moisture supply: the hydrothermal coefficient (HTC) was 1.00 (the sum of active temperatures was 1072.3 °C and the amount of precipitation was 107.1 mm). In 2015 and 2016, during the “sprouting– flowering” period, arid conditions were observed (HTC was 0.66 and 0.32, respectively). The sum of active temperatures was 1144.9–1167.9 °C, the amount of precipitation was 75.2 and 37.3 mm, respectively.

## Results

**Analysis of variation of agronomically-important traits in F_1_ hybrids.** To study the effect of cytoplasm on the combining ability of iso-nuclear CMS lines, a preliminary assessment of variation of the studied traits in 54 F_1_ hybrids was made (Table 1).

**Table 1.**
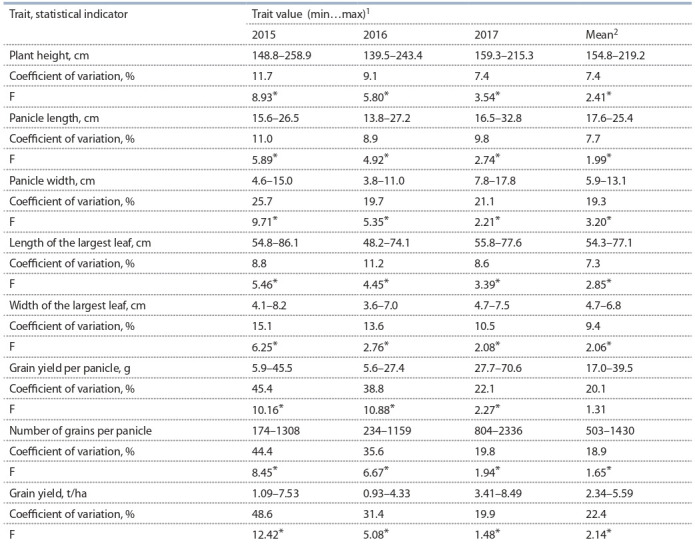
Variation of agronomically valuable traits in F_1_ hybrids obtained with iso-nucelar CMS lines with genetically different types of sterile cytoplasms (A3, A4, 9E) and nuclear genome of Zheltozernoye 10 ^1^ min and max – minimum and maximum value of the trait; ^2^ mean for 2015–2017; * p > 0.95.

The traits “plant height” (CV = 7.4–11.7 %), “inflorescence length” (CV = 7.7–11.0 %), “length of the largest leaf” (CV = 7.3–11.2 %) were characterized by low variation (see Table 1). The average variation was found for the width of the largest leaf (CV = 10.5–15.1 %), while for other traits high variation was observed. Higher coefficients of variation of the studied traits were noted in 2015, with the exception of the length of the largest leaf.

The analysis of variance confirmed the differences between the tested F_1_ hybrids for majority of agronomically valuable traits (F_observed_ > F_expected_ ). For the grain yield per panicle, on average, over three years of testing, no significant differences between hybrids were revealed at the 5 % level; therefore, the combining ability for this trait was not determined.

**Combining ability of iso-nuclear CMS lines**

**Fig. 1.**
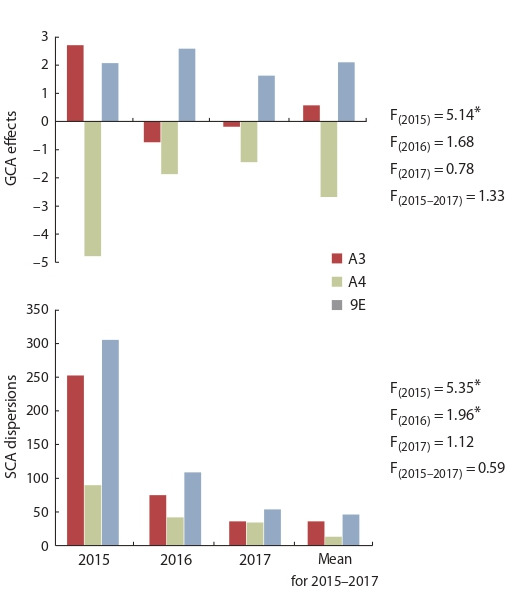
Influence of the type of sterile cytoplasm on the combining ability of iso-nuclear CMS-lines for the plant height. * p > 0.95.

**Vegetative traits.** Cytoplasms A3 and 9E significantly increased GCA effects of the CMS lines for plant height in 2015 (2.08–2.71), and SCA dispersions in 2015 (253.47–305.75), and in 2016 (75.16–109.25), in comparison with A4 cytoplasm (Fig. 1).

Differences in the effects of the GCA of the CMS lines for parameters of the largest leaf were observed only in 2016. The effects of the GCA of the CMS-line with 9E cytoplasm (1.78) were significantly higher than with CMS-line with A4 cytoplasm (–2.22). The cytoplasmic effect on the combining ability of CMS lines for the width of the largest leaf was not detected. At the same time, there is a tendency towards the manifestation of higher GCA effects of the line 9E Zheltozernoye 10 (annually). The analysis of SCA dispersion showed the influence of the CMS type on parameters of the largest leaf in 2015–2016, the A3 cytoplasm caused the most strong effect on the leaf width: SCA dispersions were 0.27–0.36. A4 cytoplasm reduced SCA dispersions according to the parameters of the largest leaf (Table 2).

**Table 2.**
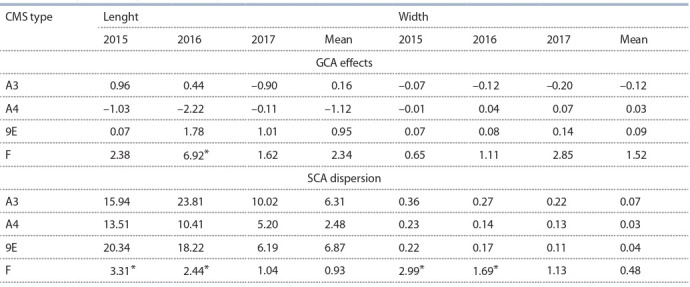
The combining ability of iso-nuclear CMS lines of sorghum Zheltozernoye 10 with genetically different types of sterile cytoplasms (A3, A4, 9E) for the parameters of the largest leaf * p > 0.95.

**Fig. 2.**
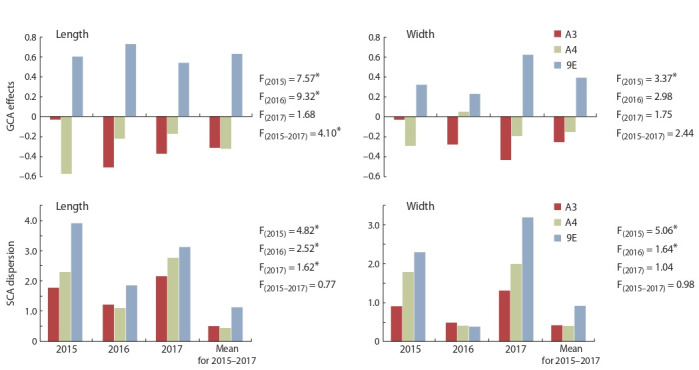
The influence of the type of cytoplasm (A3, A4, 9E) on the GCA and SCA of the iso-nuclear CMS lines of sorghum for the length and width of inflorescence. * p > 0.95.

**Generative organ traits.** A significant influence of the 9E cytoplasm on the GCA effects for the length of inflorescence was recorded in each year (Fig. 2). Higher GCA effect for the width of inflorescence was also detected in 2015 for the 9E cytoplasm: 0.32 versus –0.29 and –0.03 in the A3 and A4 cytoplasms, respectively. The dispersion of SCA for panicle parameters turned out to be significantly higher for the CMS line 9E Zh10: for the inflorescence length in each growing season, and for the inflorescence width in 2015–2016 seasons (see Fig. 2).

A stimulating cytoplasmic effect on CA of CMS lines for the panicle mass and number of grains per panicle was established in 2015–2016, i. e. under drought conditions of the cultivation of F1 hybrids. At the same time, the effects of GCA for weight and number of panicle mass were significantly higher in A3 Zh10 (1.24 and 43.19, respectively), and the SCS dispersion was lower in A4 Zh10 (in different seasons: 3.59–18.40 and 9154.16–12129.40, respectively) (Table 3).

**Table 3.**
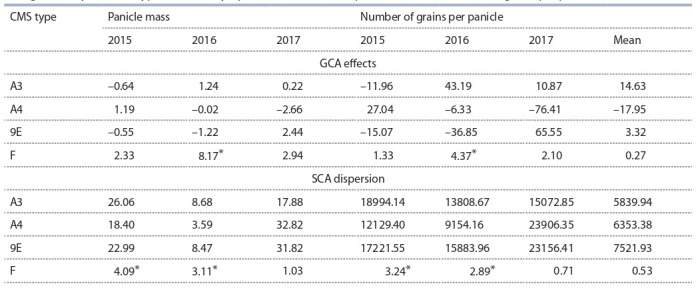
The combining ability of the iso-nuclear CMS lines of sorghum Zheltozernoye 10 with genetically different types of sterile cytoplasms (A3, A4, 9E) for panicle mass and number of grains per panicle * p > 0.95.

**Fig. 3.**
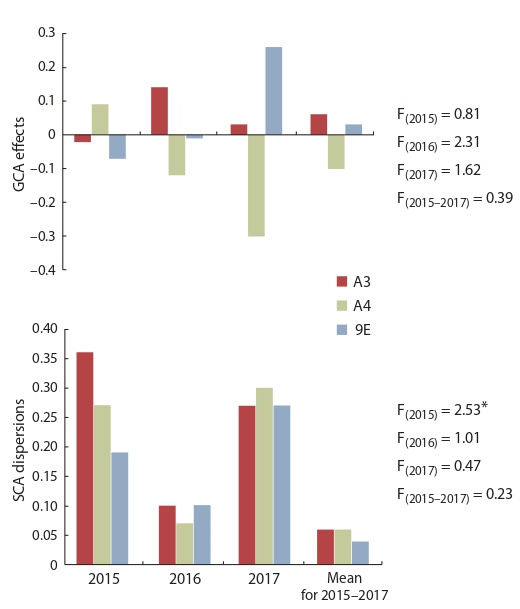
The influence of the type of cytoplasm (A3, A4, 9E) on the combining ability of iso-nuclear CMS lines of sorghum for grain yield. * p > 0.95.

The GCA effects of maternal lines for grain yield did not differ significantly (Fig. 3). On average for three-year trails, indicators of the A3 cytoplasm were slightly higher than for A4 and 9E cytoplasms (0.06 vs. –0.10 and 0.03, respective- ly). Cytoplasmic effects on SCA dispersion for grain yield were noted only in 2015: cytoplasm A3 significantly increased it in comparison with A4 and 9E cytoplasms.

## Discussion

The analysis of the combining ability of CMS lines is the most important step in sorghum hybrid breeding. One of the effective methods for analysis of CA is the topcross method. According to this method, all the studied lines are crossed with several tester lines (Kilchevsky et al., 2008). The GCA of parental line is measured by the average deviation of the trait for all hybrids with the line from the total average for all hybrids (Khotyleva et al., 2016). This method allows comparing different lines with each other, and the more testers involved in hybridization, the more accurate the results of such a comparison. In our study, iso-nuclear CMS lines that differ from each other only in the type of cytoplasm were involved in crosses. F_1_ hybrids were obtained with each of these lines, and the same lines were used as paternal parents. Therefore, a comparison the sets of F_1_ hybrids allows us to identify the presence or absence of the influence of the cytoplasm on the combining ability of the studied CMS lines.

The experimental data presented above demonstrate the effect of the cytoplasm on the CA of iso-nuclear sorghum lines. Over three years of testing, on average, a positive effect of the 9E cytoplasm on GCA for the inflorescence length (0.63) and negative effects of A3 and A4 cytoplasms (–0.32 and –0.31, respectively) on GCA for this trait were found. It should be noted that to study cytoplasmic effect on GCA for the traits determining the grain productivity of hybrids, we used panicles that set seed after free pollination. We used such approach because among the pollen parents used in our experiment, there were no CMS A3 restorers; fertility restorers of this type of CMS are extremely rare (Worstell et al., 1984; Torres-Cardona et al., 1990; Dahlberg, Madera-Torres, 1997). CMS A4 and 9E restorers were few and not capable of restoring CMS A3 fertility. Nevertheless, male-sterile hybrids grown with the free pollination regime among hundreds of thousands of fertile plants in experimental field, had 100 % seed set on all panicles of the studied hybrids. This approach has already been used previously in the study of hybrids with A3 CMS (Moran, Rooney, 2003).

It is noteworthy that the manifestation of cytoplasmic effects depends on the hydrothermal regime of plant growth. For example, significant positive effects of cytoplasms on GCA were found in dry seasons: for 9E (for the length of the largest leaf), and for A3 (for plant height), while there were no differences between them in the wet season. Remarkably, in conditions of drought, the A4 cytoplasm had a negative effect on CA for many traits (leaf length and width, number of grains per panicle, and yield). Apparently, A4 cytoplasm is less resistant to extreme drought conditions (lack of the necessary amount of precipitation, accompanied by high average daily air temperatures). As a result, the combining ability of the CMS line A4 Zheltozernoye 10 for the complex of studied traits turned out to be lower. Perhaps it is for this reason, the significance of the influence of the cytoplasm on GCA and SCA were observed only in a particular season. In addition, the manifestation of the effects of GCA is less dependent on environmental conditions than SCA. For example, CMS lines differ in the SCA for the length of the largest leaf (2015), width of the largest leaf (2015–2016), plant height (2016), panicle mass and number of grains per panicle (2015), grain yield (2015), while the effects of GCA for these traits in these seasons were not significant. A similar dependence of the manifestation of cytoplasmic effects on environmental conditions was found in pearl millet, with cytoplasms A4 and A5 showing greater environmental sustainability compared to cytoplasms A1, A2 and A3 (Chandra-Shekara et al., 2007).

According to published data, the effect of CMS type on panicle length was observed in maize hybrids (Kabanova et al., 2015); cytoplasmic effects on leaf parameters were revealed in maize hybrids with C- and S-types of CMS: hybrids with C-type CMS had higher leaf length, while S-type hybrids had higher leaf width (Frankovskaya et al., 1995).

In sorghum, the influence of the cytoplasm type on GCA for grain yield and mass of 100 grains was previously noted in the study of Indian researchers, while cytoplasm A2 had an advantage over A1 and A4 cytoplasms (Kishan, Borikar, 1989; Ramesh et al., 2006; Reddy et al., 2007, 2009). In our studies, it was found that 9E cytoplasm increased leaf width in sorghum-sudanense hybrids (Kibalnik, Elkonin, 2012). In grain sorghum hybrids this cytoplasm increased photosynthetic potential during the “heading–full maturity” period (Bychkova, Elkonin, 2016), in comparison with A3 cytoplasm. The effect of a sterile cytoplasm on the CA of sorghum CMS lines for the intensity of the initial plant growth was also found, the 9E cytoplasm contributing to an increase, and A4 cytoplasm contributing to a decrease of GCA effects (Elkonin et al., 2018). The positive effect of the 9E cytoplasm on CA for biomass productivity in dry seasons was also established (Elkonin et al., 2018), while A3 cytoplasm had a stimulating effect on grain yield in the dry and hot season (Bychkova, Elkonin, 2017). The totality of these data indicates that the cytoplasm plays a significant role in the manifestation of many agronomically valuable traits in sorghum, reducing or increasing the resistance of plants to drought stress.

## Conclusion

The effect of the cytoplasm on the combining ability of sorghum lines for a number of agronomically valuable traits (plant height, length and width of the largest leaf and of the inflorescence, panicle mass and number of grains per panicle, grain yield) was found. The manifestation of cytoplasmic effects in sorghum hybrids depends on the specific interaction of the genotypes of the parental lines and hydrothermal factors of the growing season. Significant differences in the combining ability of the iso-nuclear lines of Zh10 with the cytoplasms A3, A4 and 9E were observed during the dry seasons of vegetation (2015–2016). A3 Zh10 was distinguished by the highest GCA for the plant height, while 9E Zh10 – by the high SCA dispersion for this trait. For the length and width of the largest leaf, the highest SCA dispersion indicators are characteristic for the A3 Zh10 line. For the length and width of the inflorescence, the highest GCA effect and SCA dispersion were noted in the 9E Zh10 line. For panicle mass and the number of grains per panicle, the highest GCA effects were found in the A3 Zh10 line. The 9E Zh10 line had the highest SCA dispersion for the grain yield. A4 cytoplasm reduced combining ability for majority of the studied traits.

These experimental data can be used in grain sorghum breeding programs aimed at creating drought tolerant F_1_ hybrids with improved agronomically valuable traits.

## Conflict of interest

The authors declare no conflict of interest.
